# Psychometric properties of the Persian version of bedside teaching (BST) Instrument 

**DOI:** 10.30476/jamp.2020.88501.1343

**Published:** 2021-01

**Authors:** MOHAMMAD SAEED GHARAATI JAHROMI, MITRA AMINI, MAHSA MOOSAVI, ALIREZA SALEHI, SOMAYEH DELAVARI, ALI ASGHAR HAYAT, PARISA NABEIEI

**Affiliations:** 1 MPH Department, Shiraz Medical School, Shiraz University of Medical Sciences, Shiraz, Iran; 2 Clinical Education Research Center, Shiraz University of Medical Sciences, Shiraz, Iran; 3 Research Center for Traditional Medicine and History of Medicine, Shiraz University of Medical Sciences, Shiraz, Iran; 4 Center for Educational Research in Medical Sciences (CERMS), Department of Medical Education, School of Medicine, Iran University of Medical Sciences, Tehran, Iran

**Keywords:** Skills, Reliability, Medical students, Factor analysis, Questionnaire

## Abstract

**Introduction:**

Bedside teaching plays a crucial role in acquiring essential clinical skills. Therefore, the main aim of this study is assessing the validity and reliability of the Persian version of German bedside teaching (BST) instrument. This instrument was specially developed for evaluation of bedside teaching.

**Method:**

The present cross-sectional study was conducted on 150 last year medical students, using convenience sampling. The Persian version of the bedside teaching (BST) was used for data gathering. To calculate the reliability of the questions, Cronbach's alpha was used and to determine the construct validity of the questionnaire, confirmatory factor analysis was used. All analyses were performed in LISREL 10 and SPSS 21 software.

**Results:**

Cronbach's alpha indicated excellent reliability for each subscale (α =0.77-0.85). All of the value of the questions are more than a significant number of 1.96 and concluded to be significant. There was an acceptable fit between the hypothetical model and the data and all comparative fit indices (CFI, NFI, RFI, IFI) showed good model fitness. BST is a valid and reliable instrument for the assessment of clinical teaching at bedside. It has 18 items with 5 point Likert scales.

**Conclusion:**

The findings suggest that the Persian version of the BST questionnaire is a valid and reliable tool for the evaluation of teachers and providing feedback in a clinical setting. However, more studies should be conducted in other cities in Iran.

## Introduction

Bedside teaching (BST) is defined as discussing a disease or showing a procedure/examination at the patient's bedside by a clinical teacher ( 1 ). Sir William Osler as a model for modern medical teachers, expressed his most outstanding achievement was "[teaching] medical students in the wards, as I regard this as by far the most useful and important work I have been called upon to do" ( 2 ). He taught medical learners to "have no teaching without a patient for a text, and the best teaching is that taught by the patient himself". Bedside teaching plays a crucial role in acquiring clinical practice of skills such as history taking, physical examination, clinical reasoning, ethical decision making, empathy, instilling confidence, providing higher-order learning, professional behavior in medicine ( 3 ), and translating basic knowledge into clinical medicine ( 4 ).

Despite the undoubted benefits of BST, the frequency of clinical rounds is trending downwards ( 5 ). Concerns about unconfortability of patient in the time of case presentations at the patient bedside can lead to many clinical education programs conducted at the conference room ( 6 ). Both teachers and students encounter numerous obstacles to teach and learn in the clinical environment ( 7 ). There is a need for a validated and practical questionnaire to receive information about clinical teaching. Medical schools can give feedback or reward to teachers according to this valuable information.

There are some instruments for the assessment of clinical teaching such as SFDP26 ( 8 ), SETOC ( 9 ), FESEM ( 10 ), TRIL ( 11 ), MTEF-28 ( 12 ), UCEEM ( 13 ), MedSEQ ( 14 ). By revising all mentioned tools and two other questionnaires (SEEQ (Students Evaluations of Educational Quality [25]) and SIR II (Student Instructional Report [26]), a new instrument was created in Germany. This questionnaire was completed by medical students in Hamburg and Gottingen Medical Schools between 2014-2016. The BST7 questionnaire consists of 18 items and three factors (learning climate, clinical teaching, and preparation). Cronbach's alphas of the subscales were acceptable (0.71-0.84). It uses Likert scale (1-5 scale) to analyze the viewpoint of students. BST is a valid, reliable, and short questionnaire that specially developed for bedside teaching and also can be applied to compare medical schools ( 15 ,  16 ).

We could not find any Persian questionnaire to assess the quality of clinical teaching at academic hospitals. This study is designed to determine whether or not the Persian version of BST can be adapted for assessment of bedside teaching quality at Iranian academic hospitals.

## Methods

Based on Pearson’s article ( 17 ). A cross-sectional analytic study was conducted among 150 clinical medical students (5th, 6th, and 7th-year medicine) in 2019. BST is a valid and reliable instrument for the assessment of clinical teaching at bedside. It has 18 items with 5 point Likert scales ("strongly disagree," "disagree," "neither agree nor disagree," "agree" and "strongly agree") that measures three clinical teaching-related factors: learning climate (5 items), clinical teaching (8 items) and preparation (5 items) ( 15 ). In this study, we used the Persian translation of the BST questionnaire.

The original BST questionnaire is in German. Three professional translators translated it into Persian separately. Under the supervision of 5 educational scientists, the Persian script was written. Then backward-translation was done to check differences between the Iranian and the German versions. After a careful review, debugging contradictions, and considering cultural differences, the Persian version of the BST questionnaire was provided. Some demographic variables (sex, grade, marital status, and living place) were added to the original instrument. We calculated the number of participants by allocating five samples to each question ( 18 ). Therefore, the sample size was estimated 90 for 18 items. Due to drop-out probabilities, we increased our sample size to 150 medical students. The participants were selected by convenience sampling. All of the participants completed the BST questionnaire. Fortunately, none of our data was missed due to unanswered questions.

Cronbach's alpha was used to assess the reliability of internal consistency for each scale. The target value was considered more significant than 0.7 ( 19 ). And to check the construct validity of the questionnaire, confirmatory factor analysis was used. 

The goodness-of-fit statistics resulting from this analysis are reported. These are Root Mean Square Error of Approximation (RMSEA), Normed Fit Index (NFI), Non-Normed Fit Index (NNFI), Comparative Fit Index (CFI), Goodness-of-Fit Index (GFI) and Adjusted Goodness-of-Fit Index (AGFI). The acceptable thresholds of NFI, NNFI, CFI, GFI, and AGFI are more significant than 0.95. The value of less than 0.07 for RMSEA indicates a useful fit index. All analyses were performed in LISREL 10 software, and SPSS 21 software with a p-value of 0.05 or less considered statistically significant.

## Results

In this research, 150 medical trainees took time to complete the Persian version of the BST questionnaire. Eighty-eight of them were women (58.7%), 117 of them were single (82.4%), 71 of them were in the fifth year of the medical course (48.3%), and 85 of them were living in Shiraz (59.9%). 

**Figure 1:**
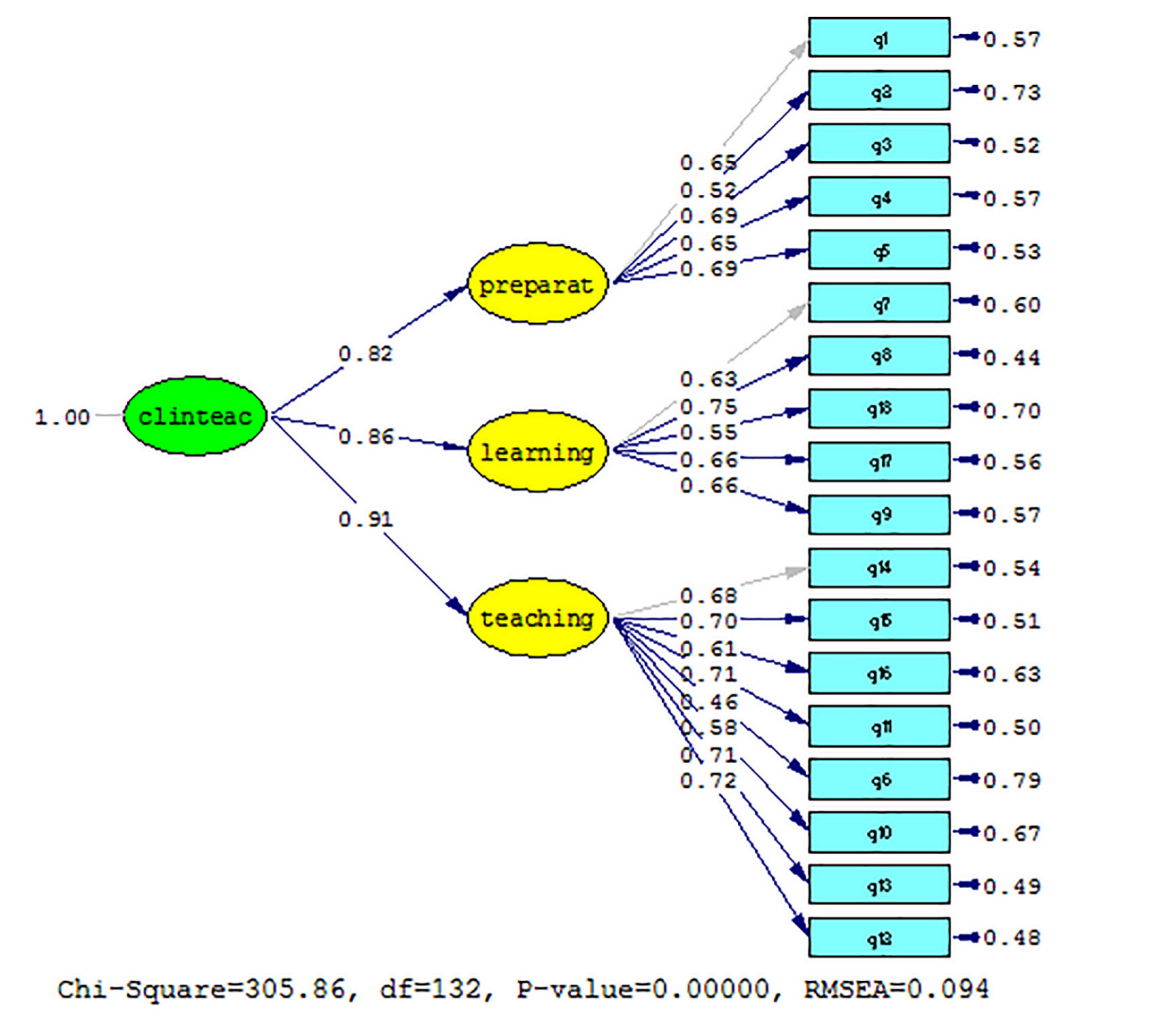
The results of Lisrel analysis for the hypothesized model for estimating standardized parameters in second-order confirmatory factor analysis.

The numbers written between latent variables (factors) and observed variables (questions) in figure 1 were factor loadings. The higher numbers of standardized factor loadings demonstrate more correlation among variables. If factor loadings were lower than 0.3, the association was weak, between 0.3 and 0.6, it was acceptable, and numbers more than 0.6 showed good correlation. (Figure 1).

**Figure 2:**
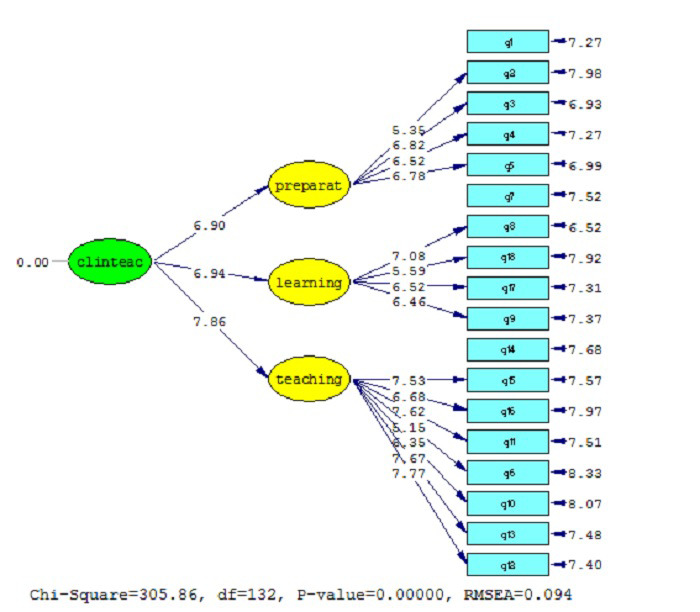
Path Diagram of Comparative Factor Analysis (CFA). Standardized coefficients

Figure 2 shows t values of standardized loadings. It will be significant if the t-value is more than 1.96 or less than -1.96. Items will have positive coefficient values if they are higher than 1.96.

Fit indices of measurement model were obtained for judging latent variables and written under the diagrams. The chi-square degrees of freedom ratio (CMIN/DF) lower than 3 reveal functional model fitness. The other indices are considered more valuable as much as numbers are closer to 1 ( 20 ).

Table 1 demonstrates fit indices that are used to assess confirmatory factor analysis. There was an acceptable fit between the hypothetical model and data, and we can see that this model is consistent with the data.

**Table 1:** Goodness of fit and the results of the Comparative Factor Analysis (CFA) for the hypothesized CFA models

Abbreviations	Full name of fits	Acceptable value	Observed value
RMSEA	Root Mean Square Error of Approximation	<0.1	0.094
CMIN/DF	Chi-degree freedom	<3	2.31
IFI	Incremental Fit Index	>=0.90	0.96
RFI	Relative Fit Index	>=0.90	0.90
NFI	Normed Fit Index	>=0.90	0.91
GFI	Goodness of Fit Index	>=0.90	0.89
AGFI	Adjusted Goodness of Fit	>=0.90	0.86
CFI	Comparative Fit Index	>=0.90	0.95

According to the results, the chi-square value is 305.86. Comparing the obtained chi-square value show that CMIN/DF is lower than three and so it is in the acceptable range. RSMEA value is 0.094. It is in the acceptable range (fair value for RMSEA is lower than 0.1) and indicates good fitness. All comparative fit indices (CFI, NFI, RFI, IFI) show good model fitness. Absolute fit indices (GFI, AFGI) were also calculated and shown in Table 2. If GFI and AFGI values are 0.90 or higher, they indicate an acceptable fitting model.

**Table 2:** Cronbach's alpha, Mean and Standard Deviation for each subscale of Bedside Teaching (BST)

Subscale	Number of Items	Cronbach's alpha	Mean (SD)
Preparation	5	0.77	13.68 (3.64)
Clinical teaching	8	0.85	23.58 (5.59)
Learning climate	5	0.77	17.14 (3.79)

The Cronbach's alpha was calculated to determine the instrument's internal consistency; it exceeded the 0.7 threshold value (0.95). Cronbach's alpha demonstrates fit indices that are used to assess confirmatory factor analysis. There was an acceptable fit between the hypothetical model and the data. 

## Discussion

In confirmatory factor analysis, we specify that the data are consistent with a certain factor structure, while in exploratory factor analysis, we try to find the latent variables and the relationship of the observed variables with them.

Clinical teachers should train the next generation of doctors and also give medical care to patients simultaneously ( 21 ). Bedside teaching, as well as patient care in clinical settings, is essential and sophisticated. All faculties need an instrument to evaluate their teachers' performances. Through a cross-cultural validation study, a reliable and validated tool is going to be available to assess bedside teaching at medical schools. This study conducted to evaluate the reliability and validity of the Persian version of the BST questionnaire.

The Cronbach's alpha reported here to each subscale was approximately similar to German studies and indicated excellent reliability ( 15 ).

The value of RSMEA indicated good fitness in the present study. It was in an acceptable range and showed good fitness among model and population covariance matrix ( 22 ).

Comparative fit index (CFI) was one of the fit indices on which the effect of sample size was minimal ( 23 ). CFI estimates fitness and makes a comparison between the sample covariance matrix and the null model. In the present study, the CFI was 0.95 and represented a good fit.

CMIN/DF ratio was lower than three, and it means that there was a little difference between the conceptual model and the observed data. 

GFI revealed the proportion of variance that was accounted for by the estimated population covariance ( 24 - 25 ); AGFI was also calculated by adjusting GFI to the degree of freedom. Both GFI and AGFI were out of an acceptable range and a little lower than 0.9. These absolute fit indices were affected by sample size and suggested using this instrument more carefully for medical students. 

The normed-fit index (NFI) was calculated to make a comparison between the χ2value of the model to the χ2 value of the null model. The amount of this statistic was more significant than 0.9 and means good fitness. In this study, the factor loadings of each indicator are substantial and indicate functional fitness among data and model.

Some significant limitations should be considered in the present research. The first limitation was that only medical students completed the BST questionnaire, and these results may not apply to other health-related disciplines such as nursing. Secondly, collecting Sample from two teaching hospitals affiliated to Shiraz University of Medical Sciences may not be representative of the whole country and Iranian culture. Therefore, more studies should be conducted in other cities in Iran. A high response rate was the main strength of this investigation.

Although the current study is based on some limitations, the findings suggest that the Persian version of the BST questionnaire has implications within the clinical setting for the evaluation of teachers and providing feedback. This instrument can be used to recognize the strengths and weaknesses of every individual trainer. Faculties also can make a decision about their employment agreements.

## Conclusion

The Persian version of bedside teaching questionnaire had good fit indices. Although this instrument has only 18 items, it can cover all important aspects of clinical teaching. This instrument can be used to evaluate clinical teachers and also provide evidence-based feedback.

## References

[1] Wojtczak A ( Med Teach 2002). ADDIN EN. REFLIST Glossary of medical education terms: Part 1.

[2] McGee S ( JAMA 2014). A piece of my mind. Bedside teaching rounds reconsidered.

[3] Carty M, O'Riordan N, Ivers M, Higgins MF ( 2020). Patient perspectives of bedside teaching in an obstetrics, Gynaecology and neonatology hospital. BMC medical education.

[4] Majdan JF, Berg KT, Schultz KL, Schaeffer A, Berg D ( 2013). Patient perceptions of bedside teaching rounds. Med Educ.

[5] Ahmed MEB (2002). What is happening to bedside clinical teaching?. Med Educ.

[6] Lehmann LS, Brancati FL, Chen MC, Roter D, Dobs AS ( 1997). The effect of bedside case presentations on patients' perceptions of their medical care. New England Journal of Medicine.

[7] Spencer J ( 2003). Learning and teaching in the clinical environment. BMJ.

[8] Litzelman DK, Stratos GA, Marriott DJ, Skeff KM ( 1998). Factorial validation of a widely disseminated educational framework for evaluating clinical teachers. Acad Med.

[9] Zuberi RW, Bordage G, Norman GR ( 2007). Validation of the SETOC instrument—student evaluation of teaching in outpatient clinics. Advances in health sciences education.

[10] Staufenbiel T ( 2000). Fragebogen zur Evaluation von universitären Lehrveranstaltungen durch Studierende und Lehrende. Diagnostica.

[11] Gollwitzer M, Schlotz W Das "Trierer Inventar zur Lehrveranstaltungsevaluation" (TRIL): Entwicklung und erste testtheoretische Erprobungen. Psychologiedidaktik und Evaluation IV: Deutscher Psychologen Verlag; 2003. p. 114-28.

[12] Beckman TJ, Lee MC, Rohren CH, Pankratz VS ( 2003). Evaluating an instrument for the peer review of inpatient teaching. Med Teach.

[13] Strand P, Sjöborg K, Stalmeijer R, Wichmann-Hansen G, Jakobsson U, Edgren G ( 2013). Development and psychometric evaluation of the undergraduate clinical education environment measure (UCEEM). Med Teach.

[14] Boyle P, Grimm M, McNeil H, Scicluna H The UNSW Medicine Student Experience Questionnaire (MedSEQ): a synopsis of its development, features and utility. Sydney: UNSW Faculty of Medicine; 2009.

[15] Dreiling K, Montano D, Poinstingl H, Müller T, Schiekirka-Schwake S, Anders S, et al ( 2017). Evaluation in undergraduate medical education: Conceptualizing and validating a novel questionnaire for assessing the quality of bedside teaching. Med Teach.

[16] Müller T, Montano D, Poinstingl H, Dreiling K, Schiekirka-Schwake S, Anders S, et al ( 2017). Evaluation of large-group lectures in medicine-development of the SETMED-L (Student Evaluation of Teaching in MEDical Lectures) questionnaire. BMC Medical Education.

[17] Pearson R, Mundfrom D ( 2010). Recommended Sample Size for Conducting Exploratory Factor Analysis on Dichotomous Data. Journal of Modern Applied Statistical Methods.

[18] Anthoine E, Moret L, Regnault A, Sébille V, Hardouin JB ( 2014). Sample size used to validate a scale: a review of publications on newly-developed patient reported outcomes measures. Health and quality of life outcomes.

[19] Ataollahi M, Amini M, Delavari S, Bazrafkan L ( 2019). Reliability and validity of the Persian version of readiness for inter-professional learning scale. International Journal of Medical Education.

[20] Afthanorhan W ( 2013). A comparison of partial least square structural equation modeling (PLS-SEM) and covariance based structural equation modeling (CB-SEM) for confirmatory factor analysis. International Journal of Engineering Science and Innovative Technology.

[21] Prideaux D, Alexander H, Bower A, Dacre J, Haist S, Jolly B, et al ( 2000). Clinical teaching: maintaining an educational role for doctors in the new health care environment. Med Educ.

[22] Byrne BM Structural equation modeling with LISREL, PRELIS, and SIMPLIS: Basic concepts, applications, and programming. UK: Informa Plc company; 2013.

[23] Fan X, Thompson B, Wang L ( 2009). Effects of sample size, estimation methods, and model specification on structural equation modeling fit indexes. Structural equation modeling: a multidisciplinary journal.

[24] Tabachnick B, Fidell LS Using multivariate statistics. 5th ed. New York. NY: Allyn and Bacon [Google Scholar]; 2007.

[25] Marsh HW ( 1982). SEEQ: a reliable, valid, and useful instrument for collecting students’ evaluations of university teaching. Br J Psychol.

